# Research on the Robustness of Focus Measure Operators Based on RRMSE

**DOI:** 10.3390/s26092748

**Published:** 2026-04-29

**Authors:** Weiying Piao, Chunxue Wang, Yongqi Han

**Affiliations:** The Higher educational Key Laboratory for Measuring & Control Technology and Instrumentation of Heilongjiang Province, Harbin University of Science and Technology, Harbin 150080, China; 2320610115@stu.hrbust.edu.cn (C.W.); 2220610178@stu.hrbust.edu.cn (Y.H.)

**Keywords:** focus measure operator, relative root mean square error, robustness

## Abstract

This paper establishes a quantitative relationship model between the relative root mean square error (*RRMSE*) and noise parameters under the additive white Gaussian noise model. According to the difference in operator operation structure, focus measure operators are divided into two categories: squared-type and absolute-value type. Theoretical derivation shows that the *RRMSE* of squared-type operators is proportional to the noise variance *σ*^2^, while for absolute-value type operators, when the noise variance is large, their *RRMSE* is approximately proportional to the noise standard deviation *σ*. On this basis, a new quantitative metric—noise response slope—is proposed to characterize the robustness of operators against noise perturbation. For squared-type operators, the value of the noise response slope can be accurately derived; for absolute-value type operators, the value of the noise response slope can only be approximated. Five squared-type operators and five absolute-value type operators are selected for experiments, and the experimental slopes are obtained via linear regression fitting. The experimental results show that for squared-type operators, the coefficient of determination exceeds 0.999; except for the sine image sequence, the relative error between the theoretical slope and the experimental slope is less than 2%, and for the sine image sequence the error is less than 10%, because the sine image has a large number of pixels whose grayscale values are close to 0 or 255. For absolute-value type operators, the coefficient of determination exceeds 0.98; there is a significant difference between the theoretical slope and the experimental slope, but the Spearman correlation coefficient between them is 1, with a two-tailed test significance level of 0.05. The proposed model can estimate the robustness of operators without adding noise to the image sequence, providing an effective analytical method for the robustness evaluation and design of focus measure operators.

## 1. Introduction

Focus measure operators are essential tools for quantifying image sharpness, and their performance directly influences the accuracy and stability of autofocus systems, microscopic imaging, and shape-from-focus [1,2,3,4,5]. In practical imaging, images are inevitably corrupted by noise, and the stability of focus measure operators under noisy conditions has become a crucial factor limiting system performance. Therefore, how to objectively and quantitatively evaluate the noise robustness of focus measure operators has been a key issue in related research.

To compare the performance of different focus measure operators, both qualitative and quantitative evaluation metrics have been proposed in the literature, and performance assessments are typically conducted from two aspects: the shape of the focus curve and its numerical characteristics [6,7,8,9]. Qualitative metrics are often used to describe macroscopic properties of the focus curve, such as unimodality, symmetry, steepness, and unbiasedness. Although these metrics can intuitively reflect the ideal shape of the curve, they lack rigorous numerical standards, making objective comparison among operators difficult. To establish a quantifiable comparison framework, early studies such as Firestone et al. introduced basic metrics like accuracy, monotonic range, and number of false maxima, but these metrics are still insufficient to fully characterize operator behavior [10]. To further improve comparability, Pertuz et al. provided a systematic review proposing metrics such as sensitivity, peak contrast, and noise robustness, which evaluate the response intensity and anti-interference ability of operators from multiple dimensions; however, their stability remains limited under strong noise or in complex real-world scenes [11]. Subsequently, Zhai et al. introduced the width of the steep region and the local extremum factor to mathematically characterize curve structure integrity and false-peak suppression capability, offering more refined evaluation means for operator selection [12]. Yu and Lu proposed a Gaussian fitting-based performance analysis method for operators, which fits the focus measure values at different focal planes to a Gaussian curve and uses parameters such as peak width and peak change rate to quantify the peak search ability and stability of operators, applicable to performance evaluation in the absence of reference images [13]. To strengthen performance assessment under noise, Piao et al. further proposed the width of the steep region (*W_s_*), steep-to-gentle ratio (*R_sg_*), peak curvature (*C_p_*), and relative root mean square error (*RRMSE*), forming a more comprehensive quantitative evaluation system [14]. Among these, *RRMSE* directly reflects the sensitivity of an operator to noise perturbation by comparing focus values before and after adding noise, providing a new approach for robustness quantification.

However, existing robustness evaluation methods, including current *RRMSE* research, are mainly applied based on experimental phenomena, and several issues deserve further exploration. First, the calculation of *RRMSE* usually depends on artificially set noise intensity, while the noise level in actual imaging systems is often unknown and changes with the environment, making the *RRMSE* value difficult to use directly for robustness comparison in different scenarios. Second, different focus evaluation operators show significant differences under the action of noise, but there is currently a lack of systematic theoretical analysis of the relationship between *RRMSE* and noise statistical parameters, which to some extent limits the interpretability and guiding significance of *RRMSE* as a robustness indicator.

To address the above issue, this paper investigates the response behavior of focus measure operators under additive white Gaussian noise (AWGN) from the perspective of noise statistical characteristics, with an emphasis on analyzing the quantitative relationship between *RRMSE* and noise parameters. Since the noise response characteristics of operators differ depending on their operation structures, the operators are first classified according to their operation structure. Then, a relationship model between *RRMSE* and noise statistical parameters is established. Based on this model, a new operator robustness metric is explored and designed. Commonly used typical focus measure operators are selected for experiments to verify the accuracy of the proposed model and the reliability of the robustness metric. This paper aims to provide a clearer theoretical basis for the robustness analysis and comparison of focus measure operators.

## 2. Methodology

### 2.1. Influence of Noise on Image Focus

The core task of focus evaluation operators is to quantify the local or global sharpness of an image through specific mathematical operations. However, in the actual imaging process, image signals are inevitably affected by noise, leading to deviations in focus evaluation results. To analyze the impact of noise on focus evaluation operators, this paper adopts the method of adding noise to image sequences and then evaluating the robustness of the focus operator by analyzing the difference between the focus evaluation values of the noisy image and the original image.

AWGN is a typical type of noise in the field of image processing. It possesses properties such as zero mean, controllable variance, and independent and identical distribution, enabling it to effectively simulate sensor thermal noise in real environments. AWGN is often used to evaluate the robustness of systems. Therefore, this paper uses the AWGN model to add noise to images. The additive noise model is expressed as follows:

(1) $$z_{x,y}=I_{x,y}+\varepsilon_{x,y}$$

where *I_x,y_* represents the original image signal, *ε_x,y_*~*N*(0,*σ*^2^) represents white Gaussian noise with a mean of 0 and variance of *σ*^2^, and *z_x,y_* represents the noisy image signal.

### 2.2. Relationship Between RRMSE and Noise Parameters

The focus values of different evaluation operators vary greatly. To quantify the impact of noise, it is necessary to design an error indicator that reflects the relative degree of noise disturbance. The Relative Root Mean Square Error (*RRMSE*) meets this requirement.

By normalizing the Root Mean Square Error (*RMSE*), it makes the error metric independent of the absolute scale of the focus values, eliminating the influence of different numerical scales across operators and enabling comparability among different operators. In focus evaluation, the *RRMSE* is computed by comparing the deviation between the noisy focus value *F_n_*(*k*) and the original focus value *F*(*k*), using the average value *μ* of the original focus values as the normalization baseline. The calculation formula is shown in Equation (2):

(2) $$\left\{\begin{array}{c} RRMSE=\frac{1}{\mu }\sqrt{\frac{1}{K}\sum_{k=1}^{K}{\left[F_{n}\left(k\right)-F\left(k\right)\right]}^{2}}\\ \mu =\frac{1}{K}\sum_{k=1}^{K}F\left(k\right) \end{array}\right.$$

where *k* represents the image number and *K* represents the total number of images. The *RRMSE* value reflects the relative interference degree of noise on the focus evaluation operator. A smaller *RRMSE* indicates that the focus value change caused by noise is relatively small and the operator is not sensitive to noise, possessing strong anti-noise robustness; a larger *RRMSE* indicates significant changes in the focus value due to noise and weak stability.

However, directly using the *RRMSE* value as a robustness indicator has limitations: it highly depends on the current noise intensity (*σ*^2^ or *σ*). Since the noise variance in actual environments is often unknown and dynamic, it is difficult to use it as an objective standard for inherent robustness. To address this, this paper extracts a feature quantity independent of noise intensity as a new robustness evaluation indicator by studying the laws of *RRMSE* changes with noise parameters.

Based on the computational structure of operators, this paper classifies them into square-type and absolute-value-type. From a signal processing perspective, the noise response mechanisms of the two types are fundamentally different:

1. Squared-type: Squaring operations amplify noise energy; noise energy is linearly added to the result. It is inferred that *RRMSE* is related to noise variance *σ*^2^.
2. Absolute-value-type: Absolute-value operations exhibit different behaviors in low-noise and high-noise regions. In the high-noise region, the output is mainly determined by noise amplitude. It is inferred that *RRMSE* is related to noise standard deviation *σ*.

Based on this theoretical hypothesis, this paper proposes using the ratio of *RRMSE* to the corresponding noise parameter to quantify the robustness of operators. Since this ratio is theoretically determined only by the operator’s structural parameters and image content characteristics, and is independent of the specific noise intensity level, it has the potential to serve as an objective evaluation metric. This means that, by calculating the theoretical ratio based on the content characteristics of the image sequence, we can directly predict and estimate the robustness of a focus operator without prior knowledge of the noise variance or additional noise-addition experiments.

### 2.3. Squared-Type Operator Derivation

According to the computational structure, square-type operators are further divided into neighborhood-difference square-type and convolution square-type.

#### 2.3.1. Neighborhood-Difference Square-Type Operators

If the operator uses the square of the difference between a central pixel and its neighboring pixels to measure focus, the focus measure value of the image can generally be simplified as:

(3) $$F=\frac{1}{MN}\sum_{x,y}\sum_{(i,j)\in \Omega }{\left(I_{x,y}-I_{i,j}\right)}^{2}$$

where Ω denotes the neighborhood of point (*x*,*y*), and *M*,*N* are the image height and width. Since image sizes are usually large, the summation can be statistically approximated by expectation:

(4) $$F\approx E\left[\sum_{(i,j)\in \Omega }{\left(I_{x,y}-I_{i,j}\right)}^{2}\right]$$

After adding white Gaussian noise, the value *F_n_* becomes:

(5) $$\begin{array}{l} F_{n}=E\left[\sum_{(i,j)\in \Omega }{\left(z_{x,y}-z_{i,j}\right)}^{2}\right]=E\left[\sum_{(i,j)\in \Omega }{\left(I_{x,y}-I_{i,j}+\varepsilon_{x,y}-\varepsilon_{i,j}\right)}^{2}\right]\\ =E\left[\sum_{(i,j)\in \Omega }{\left(I_{x,y}-I_{i,j}\right)}^{2}\right]+2E\left[\sum_{(i,j)\in \Omega }\left(I_{x,y}-I_{i,j}\right)\left(\varepsilon_{x,y}-\varepsilon_{i,j}\right)\right]+E\left[\sum_{(i,j)\in \Omega }{\left(\varepsilon_{x,y}-\varepsilon_{i,j}\right)}^{2}\right] \end{array}$$

Because the noise is spatially independent and zero-mean, the difference in two noise terms (*ε_x_*_,*y*_−*ε_i_*_,*j*_) still follows a zero-mean Gaussian distribution with variance 2*σ*^2^. In expectation, the cross term between signal and noise is zero, so the effect of noise on the focus measure mainly comes from the square term of the noise difference:

(6) $$F_{n}-F=E\left[\sum_{(i,j)\in \Omega }{\left(\varepsilon_{x,y}-\varepsilon_{i,j}\right)}^{2}\right]=2n\sigma^{2}$$

where *n* represents the number of difference operations in the neighborhood Ω. Substituting this into the definition of *RRMSE* yields:

(7) $$RRMSE=\frac{2n}{\mu }\sigma^{2}$$

Thus, for neighborhood-difference square-type operators, *RRMSE* is approximately proportional to the noise variance *σ^2^*. The coefficient before *σ^2^* quantitatively reflects the growth rate of *RRMSE* with noise variance; we call it the noise response slope. Its value mainly depends on the ratio *n/μ*. Note that the noise response slope is not a fixed constant: as the number of participating neighborhood points *n* increases, the expectation of the focus measure *μ* also increases accordingly. Therefore, the noise response slope is an inherent property determined jointly by the operator structural parameter *n* and the image characteristic *μ*.

#### 2.3.2. Convolution Square-Type Operators

If the operator convolves the image with a mask and then squares the convolution result to measure focus, the focus measure value *F* can generally be simplified as:

(8) $$F=\frac{1}{MN}\sum_{x,y}\left[{\left(H*I\right)}_{x,y}^{2}\right]=\frac{1}{MN}\sum_{x,y}{\left[\sum_{u,v}H\left(u,v\right)I\left(x-u,y-v\right)\right]}^{2}$$

where *H* denotes the mask and ∗ denotes convolution. Under AWGN, because convolution is a linear operation, the noise after convolution still follows a zero-mean Gaussian distribution, with variance determined by the noise variance and the mask coefficients. Then, the focus measure under noise, *F_n_* is:

(9) $$\begin{array}{l} F_{n}=E\left[{\left(H*z\right)}_{x,y}^{2}\right]=E\left\{{\left[{\left(H*I\right)}_{x,y}+{\left(H*\varepsilon \right)}_{x,y}\right]}^{2}\right\}\\ =E\left[{\left(H*I\right)}_{x,y}^{2}\right]+2E\left[{\left(H*I\right)}_{x,y}{\left(H*\varepsilon \right)}_{x,y}\right]+E\left[{\left(H*\varepsilon \right)}_{x,y}^{2}\right] \end{array}$$

Since (*H*∗*ε*)*_x,y_* has zero mean, the expectation of the cross term in the square expansion is zero, and the error is dominated by the square of the noise convolution term:

(10) $$\begin{array}{lll} F_{n}-F & \approx & E\left[{\left(H*\varepsilon \right)}_{x,y}^{2}\right]=D\left[{\left(H*\varepsilon \right)}_{x,y}\right]\\ & = & D\left[\sum_{u,v}H\left(u,v\right)\varepsilon \left(x-u,y-v\right)\right]\\ & = & \sum_{u,v}H^{2}\left(u,v\right)D\left[\varepsilon \left(x-u,y-v\right)\right]\\ & = & \sigma^{2}\sum_{u,v}H^{2}\left(u,v\right) \end{array}$$

Inserting this into the *RRMSE* definition gives:

(11) $$RRMSE=\frac{\sigma^{2}}{\mu }\left[\sum_{u,v}H^{2}\left(u,v\right)\right]$$

Therefore, for convolution square-type operators, *RRMSE* is also proportional to noise variance *σ*^2^; only the expression of the noise response slope differs from that of neighborhood-difference square-type operators. The key factors determining the noise response slope include not only the image mean *μ* but also the sum of squares of the convolution kernel coefficients (the energy of the filter). This implies that when designing an operator, by rationally optimizing the energy distribution of the mask coefficients, the noise robustness can be theoretically predicted.

From the above derivations, both neighborhood-difference and convolution square-type operators have *RRMSE* proportional to *σ*^2^; therefore, they can be uniformly classified as square-type operators.

### 2.4. Absolute-Value-Type Operator Derivation

Based on the computational structure, absolute-value-type operators are further divided into neighborhood-difference absolute-value type and convolution absolute-value type.

#### 2.4.1. Neighborhood-Difference Absolute-Value-Type Operators

If the operator uses the absolute value of the difference between a central pixel and its neighboring pixels to measure focus, the focus measure value *F* can generally be simplified as:

(12) $$F=\frac{1}{MN}\sum_{x,y}\sum_{(i,j)\in \Omega }\left|I_{x,y}-I_{i,j}\right|=E\left[\sum_{(i,j)\in \Omega }\left|I_{x,y}-I_{i,j}\right|\right]$$

*F_n_* denote the focus measure after adding noise:

(13) $$F_{n}=\frac{1}{MN}\sum_{x,y}\sum_{(i,j)\in \Omega }\left|z_{x,y}-z_{i,j}\right|=E\left[\sum_{(i,j)\in \Omega }\left|I_{x,y}-I_{i,j}+\varepsilon_{x,y}-\varepsilon_{i,j}\right|\right]$$

Any image can be divided into grayscale flat areas, grayscale change areas, and grayscale transition areas. If the grayscale variance of a certain area is much smaller than the noise variance, then this area belongs to a grayscale flat area. If the grayscale variance is much greater than the noise variance, then this area belongs to a region with obvious grayscale variation. If the grayscale variance is close to the noise variance, then this region belongs to a grayscale transition region. This paper mainly discusses the influence of grayscale flat areas and grayscale change areas on the output of the focusing measure operator. For the grayscale transition areas, this paper does not discuss the resulting error, which will be explained by experiments later.

Let *F*′ be the focus measure from flat regions of the original image, $F_{n}^{\prime }$ be the focus measure from flat regions after adding noise, and *P* be the number of pixels in flat regions. When the noise variance is large, it can be assumed that *I_x_*_,*y*_−*I_i_*_,j_ << *ε_x_*_,*y*_−*ε_i_*_,*j*;_ therefore,

(14) $$\begin{array}{lll} F_{n}^{\prime } & \approx & \frac{1}{P}\sum_{x,y}\sum_{(i,j)\in \Omega }\left[\left|\varepsilon_{x,y}-\varepsilon_{i,j}\right|+\mathrm{sgn}\left(I_{x,y}-I_{i,j}\right)\left|I_{x,y}-I_{i,j}\right|\right]\\ & = & E\left[\sum_{(i,j)\in \Omega }\left|\varepsilon_{x,y}-\varepsilon_{i,j}\right|\right]+E\left[\sum_{(i,j)\in \Omega }\mathrm{sgn}\left(I_{x,y}-I_{i,j}\right)\left|I_{x,y}-I_{i,j}\right|\right] \end{array}$$

(15) $$F_{n}^{\prime }-F^{\prime }\approx \left\{\begin{array}{l} E\left[\sum_{(i,j)\in \Omega }\left|\varepsilon_{x,y}-\varepsilon_{i,j}\right|\right],\mathrm{sgn}\left(I_{x,y}-I_{i,j}\right)\geq 0\\ E\left[\sum_{(i,j)\in \Omega }\left|\varepsilon_{x,y}-\varepsilon_{i,j}\right|\right]-2E\left[\sum_{(i,j)\in \Omega }\left|I_{x,y}-I_{i,j}\right|\right],\mathrm{sgn}\left(I_{x,y}-I_{i,j}\right)<0 \end{array}\right.$$

Let $C=E\left[\sum_{(i,j)\in \Omega }\left|I_{x,y}-I_{i,j}\right|\right]$. Note that the probabilities of sgn(*I_x_*_,*y*_−*I_i_*_,*j*_) ≥ 0 and sgn(*I_x_*_,*y*_−*I_i_*_,*j*_) < 0 are approximately equal, so there is

(16) $$F_{n}^{\prime }-F^{\prime }\approx E\left[\sum_{(i,j)\in \Omega }\left|\varepsilon_{x,y}-\varepsilon_{i,j}\right|\right]-C=\frac{2\sigma n}{\sqrt{\pi }}-C$$

For regions with significant gray variation, it can be assumed that *I_x_*_,*y*_−*I_i_*_,j_ >> *ε_x_*_,*y*_−*ε_i_*_,j_. Let *F*″ be the focus measure from those regions in the original image, $F_{n}^{\prime \prime }$ be the focus measure after adding noise, and *Q* be the number of pixels in those regions. Then,

(17) $$\begin{array}{ccc} F_{n}^{\prime \prime } & \approx & \frac{1}{Q}\sum_{x,y}\sum_{(i,j)\in \Omega }\left[\left|I_{x,y}-I_{i,j}\right|+\mathrm{sgn}\left(\varepsilon_{x,y}-\varepsilon_{i,j}\right)\left|\varepsilon_{x,y}-\varepsilon_{i,j}\right|\right]\\ & = & E\left[\sum_{(i,j)\in \Omega }\left|I_{x,y}-I_{i,j}\right|\right]+E\left[\sum_{(i,j)\in \Omega }\mathrm{sgn}\left(\varepsilon_{x,y}-\varepsilon_{i,j}\right)\left|\varepsilon_{x,y}-\varepsilon_{i,j}\right|\right] \end{array}$$

(18) $$F_{n}^{\prime \prime }-F^{\prime \prime }\approx E\left[\sum_{(i,j)\in \Omega }\mathrm{sgn}\left(\varepsilon_{x,y}-\varepsilon_{i,j}\right)\left|\varepsilon_{x,y}-\varepsilon_{i,j}\right|\right]$$

Considering that the probabilities of sgn(ε*_x_*_,*y*_−*ε_i_*_,*j*_) ≥ 0 and sgn(ε*_x_*_,*y*_−*ε_i_*_,*j*_) < 0 are approximately equal; therefore, for areas where the grayscale of the image changes significantly, it can be considered that $F_{n}^{\prime \prime }$ − *F*″ ≈ 0.

For any image, without considering gray transition regions, the above two cases can be obtained:

(19) $$F_{n}-F\approx \frac{P}{MN}\left[F_{n}^{\prime }-F^{\prime }\right]=\frac{P}{MN}\left[\frac{2n\sigma }{\sqrt{\pi }}-C\right]$$

Now,

(20) $$RRMSE\approx \frac{P}{\mu MN}\left(\frac{2n\sigma }{\sqrt{\pi }}-C\right)$$

It can be seen from Equation (20) that, under the condition of a constant area of grayscale flat areas, the *RRMSE* of the neighborhood difference absolute-value type operator is proportional to the noise standard deviation *σ*. In practical applications, the size of the grayscale flat areas correlates with *σ*^2^ and increases with its magnitude. When the area of grayscale flat areas does not change significantly, the *RRMSE* can be considered approximately proportional to the noise standard deviation *σ*, and the noise response slope can be used to represent the robustness of the operator. *C* reflects the intercept of the linear relationship, which is always negative. Experimental results show that the value of C is generally small and has little effect on robustness evaluation; therefore, in practical applications, the influence of *C* can be ignored.

Since the area of grayscale flat areas in an image is generally unknown, the actual value of the noise response slope of absolute-value type operator is difficult to obtain accurately. To estimate the noise response slope, we can set *P*/*MN* = 1, meaning the area of grayscale flat areas equals the total image area. The noise response slope obtained under this condition is referred to as the theoretical slope in this paper. Obviously, the theoretical slope is always greater than the actual value of the noise response slope and can be considered as the theoretical upper bound of the actual value.

#### 2.4.2. Convolution Absolute-Value-Type Operators

If the operator convolves the image with a mask and then takes the absolute value of the convolution result, the focus measure *F* can generally be simplified as:

(21) $$F=\frac{1}{MN}\sum_{x,y}\left|{\left(H*I\right)}_{x,y}\right|=E\left[\left|{\left(H*I\right)}_{x,y}\right|\right]$$

The focus measure of the noisy image *F_n_* is:

(22) $$F_{n}=E\left[\left|{\left(H*z\right)}_{x,y}\right|\right]=E\left[\left|{\left(H*I\right)}_{x,y}+{\left(H*\varepsilon \right)}_{x,y}\right|\right]$$

Again, we separate the image into flat regions and regions with significant variation. For flat regions, it can be assumed that (*H*∗*I*)*_x_*_,*y*_ << (*H*∗*ε*)*_x_*_,*y*;_ therefore,

(23) $$F_{n}^{\prime }\approx E\left[\left|{\left(H*\varepsilon \right)}_{x,y}\right|+\mathrm{sgn}{\left(H*I\right)}_{x,y}\left|{\left(H*I\right)}_{x,y}\right|\right]$$

At this moment,

(24) $$F_{n}^{\prime }-F^{\prime }\approx \left\{\begin{array}{l} E\left[\left|{\left(H*\varepsilon \right)}_{x,y}\right|\right]\begin{array}{cc} , & \mathrm{sgn}{\left(H*I\right)}_{x,y}\geq 0 \end{array}\\ E\left[\left|{\left(H*\varepsilon \right)}_{x,y}\right|\right]-2E\left[\left|{\left(H*I\right)}_{x,y}\right|\right]\begin{array}{cc} , & \mathrm{sgn}{\left(H*I\right)}_{x,y}<0 \end{array} \end{array}\right.$$

Let *C* = *E*[∣(*H*∗*I*)*_x,y_*∣], considering that the probabilities of sgn(*H*∗*I*)*_x_*_,*y*_ ≥ 0 and sgn(*H*∗*I*)*_x_*_,*y*_ < 0 are approximately equal, so that

(25) $$F_{n}^{\prime }-F^{\prime }\approx E\left[\left|{\left(H*\varepsilon \right)}_{x,y}\right|\right]-C$$

For regions with significant variation, it can be assumed that (*H*∗*I*)*_x_*_,*y*_ >> (*H*∗*ε*)*_x_*_,*y*;_ therefore, $F_{n}^{\prime \prime }$ − *F*″ ≈ 0. Combining both cases yields:

(26) $$F_{n}-F=\frac{P}{MN}\left\{E\left[\left|{\left(H*\varepsilon \right)}_{x,y}\right|\right]-C\right\}$$

We know that

(27) $$E\left[\left|{\left(H*\varepsilon \right)}_{x,y}\right|\right]=\sigma \sqrt{\frac{2}{\pi }\sum_{u,v}H^{2}(u,v)}$$

We obtain

(28) $$RRMSE\approx \frac{P}{\mu MN}\left[\sigma \sqrt{\frac{2}{\pi }\sum_{u,v}H^{2}(u,v)}-C\right]$$

Equation (28) demonstrates that the relationship between the *RRMSE* of convolutional absolute-value operators and *σ* resembles that of neighborhood difference absolute-value operators. Thus, their robustness can still be quantified using the noise response slope.

From the above derivations, both neighborhood-difference and convolution absolute-value-type operators have *RRMSE* approximately proportional to *σ*; thus, they can be uniformly classified as absolute-value-type operators.

### 2.5. Summary

Combining the theoretical derivations in Section 2.3 and Section 2.4, although square-type and absolute-value-type operators have different computational forms, their noise response behavior under AWGN can be unified into a linear model in a statistical sense. The only difference is the independent variable of the linear model, which is determined by the operator structure: square-type operators correspond to noise variance *σ*^2^, while absolute-value-type operators correspond to noise standard deviation *σ*.

Based on this unified description, we introduce the noise response slope (*k_theory_*) to characterize the inherent sensitivity of a focus measure operator to noise perturbation. It is defined as the ratio of *RRMSE* to the corresponding noise parameter, i.e., the theoretical slope of the *RRMSE*–noise relationship curve, as shown in Equation (29). This parameter integrates the operator’s structural parameters, image statistics, and noise propagation behavior into a single scalar indicator, providing a unified quantitative standard for comparing the robustness of different operators.

(29) $$k_{theory}\approx RRMSE/\psi_{noise}$$

where *Ψ_noise_ *denotes the noise statistical parameter matching the operator type (*σ*^2^ for square-type, *σ* for absolute-value-type). Table 1 lists the expressions of *k_theory_ *for different operator types. The establishment of this unified noise response model clarifies the physical meaning of *k_theory_* and provides a direct basis for verifying the theoretical analysis by fitting the *RRMSE*–noise curve slopes in subsequent experiments.

## 3. Focus Measure Operators

To verify the theoretical differences in noise response between square-type and absolute-value-type operators [15,16,17], this section introduces the typical operators selected for the experiments.

### 3.1. Square-Type Focus Operators

1. Brenner

Brenner operator measures image detail intensity by calculating the square of the gray difference between two pixels spaced horizontally by two. It is defined as:

(30) $$F=\sum_{x,y}[I(x,y)-I(x+2,y){]}^{2}$$

2. EOG

Energy of Gradient operator uses the cumulative sum of squared gray differences between adjacent pixels in both horizontal and vertical directions as the sharpness measure.

(31) $$F=\sum_{x,y}{\left[I\left(x+1,y\right)-I\left(x,y\right)\right]}^{2}+{\left[I\left(x,y+1\right)-I\left(x,y\right)\right]}^{2}$$

3. Roberts

Roberts operator adopts the squared sum of the cross subtraction of the grayscale values of four adjacent pixels as the gradient value at each pixel. The gradient values of all pixels are then summed to obtain the measure.

(32) $$F=\sum_{x}\sum_{y}\left\{{\left[I\left(x+1,y+1\right)-I\left(x,y\right)\right]}^{2}+{\left[I\left(x+1,y\right)-I\left(x,y+1\right)\right]}^{2}\right\}$$

4. Tenengrad

Tenengrad operator is based on the Sobel operator. It calculates the sum of squares of the horizontal and vertical gradient responses at each pixel to represent the overall gradient energy of the image.

(33) $$F=\sum_{x,y}\left[{G_{x}}^{2}(x,y)+{G_{y}}^{2}(x,y)\right]$$

(34) $$G_{x}=\left(\begin{array}{ccc} 1 & 0 & -1\\ 2 & 0 & -2\\ 1 & 0 & -1 \end{array}\right)*I(x,y)\;\;\;\;\;G_{y}=\left(\begin{array}{ccc} 1 & 2 & 1\\ 0 & 0 & 0\\ -1 & -2 & -1 \end{array}\right)*I(x,y)$$

*G_x_* and *G_y_* are the layer responses of the Sobel operator in the horizontal and vertical directions.

5. EOL

Energy of Laplacian operator uses the sum of squares of the Laplacian operator response. The Laplacian operator is more sensitive to details and edges, capturing local variations better.

(35) $$F=\sum_{x,y}{\left[I\left(x+1,y\right)+I\left(x-1,y\right)+I\left(x,y+1\right)+I\left(x,y-1\right)-4I\left(x,y\right)\right]}^{2}$$

Among these five operators, Brenner, EOG, and Roberts belong to the neighborhood-difference square-type, while Tenengrad and EOL belong to the convolution square-type.

### 3.2. Absolute-Value-Type Operators

1. SMD

Sum of Modulus of Differences operator computes the sum of absolute gray differences between adjacent pixels to reflect the intensity of gray variations. It has low computational cost and good real-time performance.

(36) $$F=\sum_{x,y}\left[\left|I\left(x+1,y\right)-I\left(x,y\right)\right|+\left|I\left(x,y+1\right)-I\left(x,y\right)\right|\right]$$

2. Improved Brenner

Improved Brenner operator adds vertical gradient calculation to the traditional Brenner algorithm and uses absolute summation instead of squares, making it more adaptable to complex images while maintaining computational efficiency [18].

(37) $$F=\sum_{x,y}\left[\left|f\left(x+2,y\right)-f\left(x,y\right)\right|+\left|f\left(x,y+2\right)-f\left(x,y\right)\right|\right]$$

3. RobertsAbs

RobertsAbs is an alternative form of the Roberts operator, directly using the sum of absolute values of the two gradient components obtained from the Roberts cross operator. In fact, many FM operators have both absolute and square versions.

(38) $$F=\sum_{x,y}\left[\left|I\left(x,y\right)-I\left(x+1,y+1\right)\right|+\left|I\left(x+1,y\right)-I\left(x,y+1\right)\right|\right]$$

4. SML

Sum of Modified Laplacian operator accumulates the absolute values of horizontal and vertical second-order differences, highlighting details while being relatively robust.

(39) $$F=\sum_{x,y}\left[\left|2I\left(x,y\right)-I\left(x-1,y\right)-I\left(x+1,y\right)\right|+\left|2I\left(x,y\right)-I\left(x,y-1\right)-I\left(x,y+1\right)\right|\right]$$

5. Prewitt

Prewitt operator uses the Prewitt operator to compute the sum of absolute horizontal and vertical gradients.

(40) $$F=\sum_{x,y}\left[\left|P_{x}(x,y)\right|+\left|P_{y}(x,y)\right|\right]$$

(41) $$P_{x}=\left(\begin{array}{ccc} -1 & 0 & 1\\ -1 & 0 & 1\\ -1 & 0 & 1 \end{array}\right)*I(x,y)\;\;\;\;\;P_{y}=\left(\begin{array}{ccc} -1 & -1 & -1\\ 0 & 0 & 0\\ 1 & 1 & 1 \end{array}\right)*I(x,y)$$

*P_x_* and *P_y_* are the layer responses of the Prewitt operator in the horizontal and vertical directions.

Among these five operators, SMD, Improved Brenner, and RobertsAbs belong to the neighborhood-difference absolute-value-type, while SML and Prewitt belong to the convolution absolute-value-type.

## 4. Experiments

### 4.1. Experimental Setup

To comprehensively evaluate the accuracy and applicability of the proposed theoretical model, the experiments were designed to cover diverse test data ranging from ideal simulations to complex real-world scenarios. The experimental study mainly consisted of two parts: first, verifying the linear assumption of the noise response model; and second, validating the effectiveness of the theoretical sensitivity coefficient as a robustness indicator.

#### 4.1.1. Experimental Datasets

**Figure 1:**
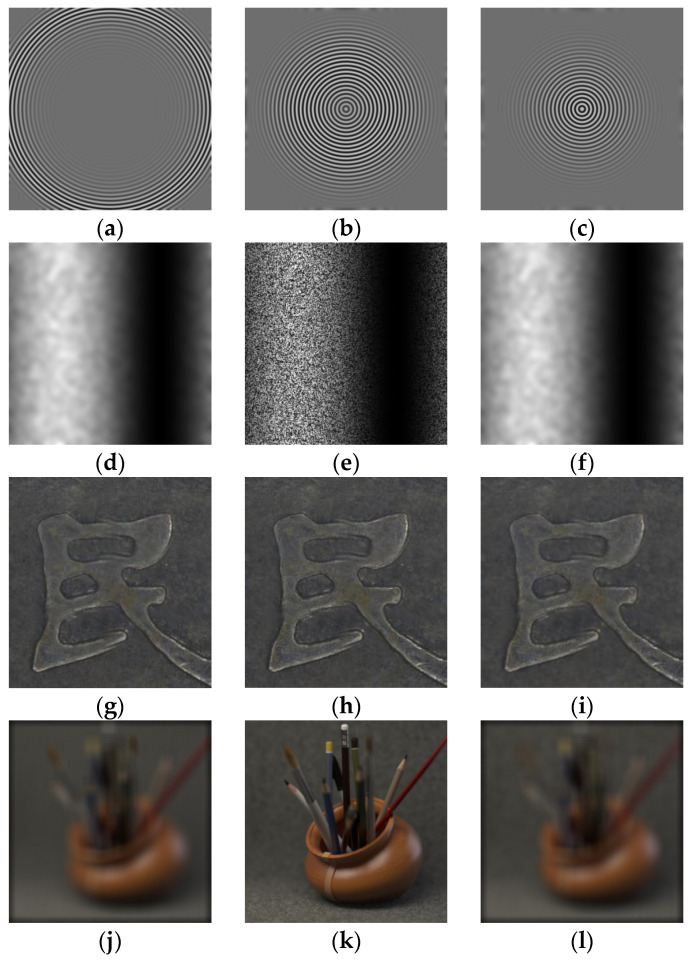
Examples of image sequences: (**a**–**c**) Cone; (**d**–**f**) Sine; (**g**–**i**) Coin; (**j**–**l**) Pencil.

Two representative types of image sequences are selected for the experiments to systematically evaluate the validity and applicability of the proposed theoretical model under both controlled conditions and real-world scenes. Each sequence covers multiple images at different depths of field. Partial examples are shown in Figure 1, and the sequence parameters are listed in Table 2.

The first category consists of simulated sequences, generated according to the defocus imaging model in [19], including two typical surface topographies: a cone and a sinusoidal curved surface. This type of data features precisely controllable focal plane variations and structural parameters, effectively excluding complex interfering factors present in real imaging, thereby enabling validation of the correctness and underlying mechanisms of the theoretical model. Among them, the cone and the sinusoidal curved surface represent monotonic and periodic structures, respectively, covering typical geometric characteristics.

The second category comprises real sequences, all sourced from public datasets, including a coin surface and a pencil image sequence. This type of data exhibits complex texture distributions and rich spectral components, which can be used to evaluate the model’s performance under actual imaging conditions, thereby verifying its generalization capability and engineering applicability.

The experiment simulated the noise environment by adding zero-mean AWGN. For squared operators, the noise variance *σ*^2^ was set within the range of [0,100]; for absolute-value operators, the noise standard deviation *σ* was set within the range of [0,10].

To simulate the dynamic range limitations of real imaging systems and evaluate their impact, this experiment established two grayscale processing conditions for comparison:

Restricted grayscale range: constraining the grayscale values of noise-embedded images within the 0–255 range to approximate real-world noise constraints, enabling more objective assessment of the model’s actual performance in practical applications.

Unrestricted grayscale range: Allows noise-superimposed grayscale values to exceed the 0–255 range to maintain the ideal statistical distribution of noise.

Under each of the above conditions, to eliminate the influence of random noise and improve the statistical reliability, multiple independent repeated experiments are conducted for each noise intensity. In each experiment, independent Gaussian white noise is randomly generated and added to the original image sequence. For each operator, the experiment is repeated 5 times at each noise level, and the corresponding *RRMSE*–noise relationship curves and their linear fitting results are calculated.

#### 4.1.2. Evaluation Metrics

To quantitatively evaluate the effectiveness of the established theoretical model, this paper selects Linear goodness of fit and relative error as the core evaluation indicators. This selection is based on the structural characteristics of the theoretical model and the validation objectives, as detailed below:

1. Linear goodness of fit

The core assumption of the theoretical model in this paper is that a linear relationship exists between the RRMSE and the noise parameter (*σ*^2^ or *σ*). Therefore, the primary issue in verifying the model is to determine whether the experimental data conform to a linear trend. The linear goodness of fit, *R*^2^, can comprehensively measure the consistency between the experimental data and the theoretical linear model. When *R*^2^ is close to 1, it indicates that the experimental results statistically support the proposed linear relationship. Thus, this metric is used to verify the rationality of the model’s structural assumption. The definition of *R*^2^ is shown in Equation (42):

(42) $$R^{2}=1-\frac{\sum_{i=1}^{N}{\left(y_{i}-{\hat{y}}_{i}\right)}^{2}}{\sum_{i=1}^{N}{\left(y_{i}-{\bar{y}}_{i}\right)}^{2}}$$

where $y_{i}$ represents the experimental observed values, ${\hat{y}}_{i}$ the linear fitted values, $\bar{y}$ the mean of the observed values, and *N* the number of data points.

2. Relative error

After confirming the validity of the linear relationship, it is further necessary to evaluate the reliability of the noise response slope as a robustness indicator. To this end, a relative error between the theoretical slope *k_theory_* and the experimental regression slope *k_exp_* is introduced to measure the predictive accuracy of the theoretical noise response slope value relative to the actual value. This relative error is denoted by Re and is defined as shown in Equation (43).

(43) Re=kexp−ktheoryktheory×100%

The experiments were conducted on the Microsoft Windows 11 (Microsoft Corp., Redmond, WA, USA), with an Intel Core i5-1035G1 processor (Intel Corporation, Santa Clara, CA, USA) and 8 GB of memory. MATLAB R2017 (The MathWorks, Inc., Natick, MA, USA) was used as the software platform.

### 4.2. Linear Validation of the Noise Response Model

This section focuses on validating the unified noise response model derived in Section 2. Specifically, for squared-type operators, the *RRMSE* is proportional to the noise variance *σ*^2^; for absolute-value-type operators, the *RRMSE* is proportional to the noise standard deviation *σ* in the noise-dominant region.

#### 4.2.1. Linear Validation for Squared-Type Operators

**Figure 2:**
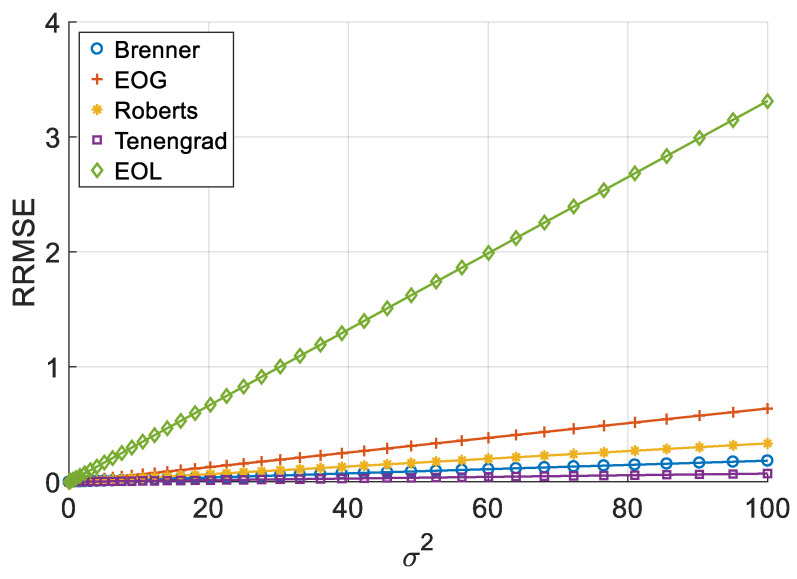
*RRMSE*–*σ*^2^ experimental values and their fitted lines under the Cone sequence.

**Figure 3:**
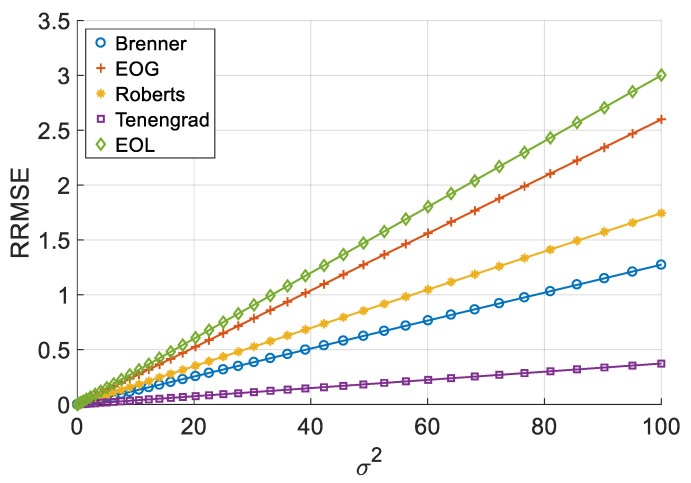
*RRMSE*–*σ*^2^ experimental values and their fitted lines under the Sine sequence.

**Figure 4:**
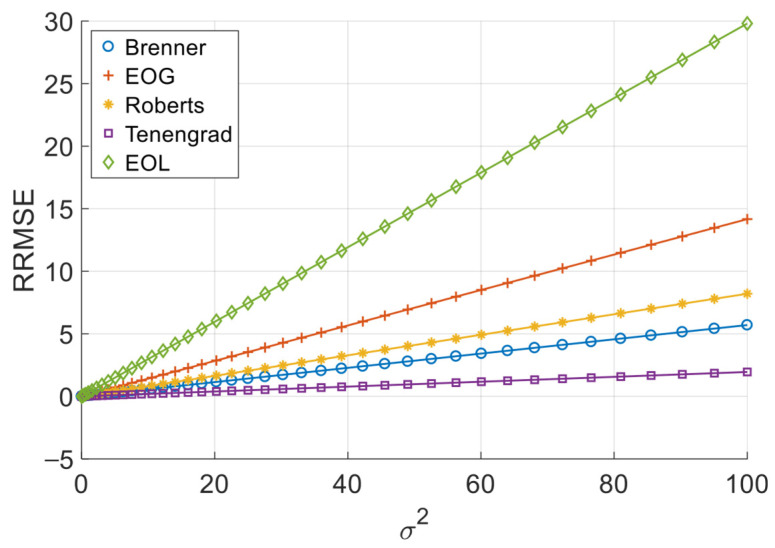
*RRMSE*–*σ*^2^ experimental values and their fitted lines under the Coin sequence.

**Figure 5:**
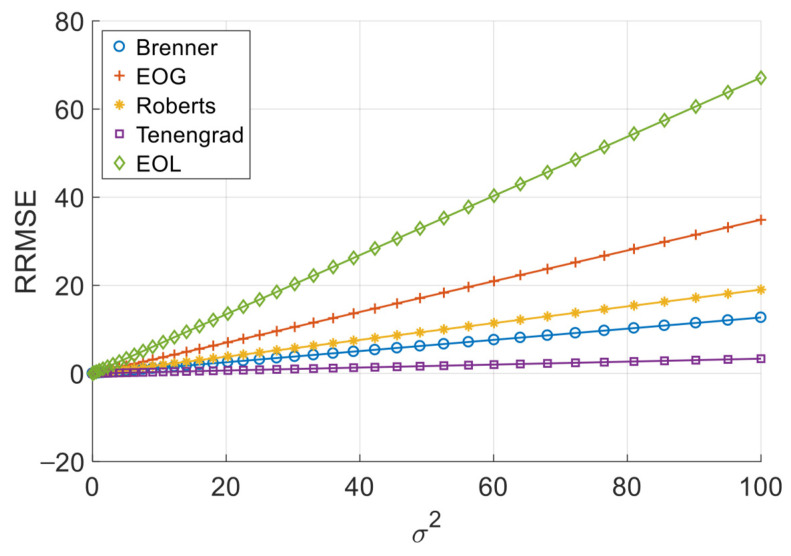
*RRMSE*–*σ*^2^ experimental values and their fitted lines under the Pencil sequence.

Figure 2, Figure 3, Figure 4 and Figure 5 illustrate the single-experiment values and fitted lines of *RRMSE* versus *σ*^2^ for the five squared-type operators introduced in Chapter 3 under different image sequences.

As shown in Figure 2, Figure 3, Figure 4 and Figure 5, for all four image sequences, the *RRMSE* of each squared-type focus measure operator exhibits an approximately linear increasing trend with the noise variance *σ*^2^. To quantitatively evaluate the reliability of this linear relationship, Table 3 presents the coefficient of determination *R*^2^ for each operator on each image sequence.

The experimental results show that whether for simulated image sequences (Cone and Sine) or real image sequences (Coin and Pencil), the *R*^2^ values of all squared-type operators remain above 0.999, indicating a strong linear correlation between RRMSE and *σ*^2^ within the considered noise range. Although slight differences in *R*^2^ values exist across image sequences, all values remain at a very high level, demonstrating that this linear relationship is highly stable. This observation is consistent with the theoretical analysis of squared-type operators presented in Section 2.3, namely that under additive Gaussian noise, the *RRMSE* of squared-type operators is linearly related to the noise variance.

Table 4, Table 5, Table 6 and Table 7 present, for five squared-type operators on different image sequences, the theoretical slope *k_theory_*, as well as the mean values of the experimental slopes *k*_*exp*1_ (Restricted grayscale range) and *k*_*exp*2_ (Unrestricted grayscale range) obtained from repeated experiments under the two grayscale processing conditions, together with the mean and standard deviation of the corresponding relative errors Re_1_ and Re_2_. Due to the minimal variations in *k*_*exp*1_ and *k*_*exp*2_ during experiments, resulting in small standard deviations, this study only presents the standard deviations *S*_1_ and *S*_2_ for Re_1_ and Re_2_. The numbers in parentheses within the table indicate the slope-based operator robustness ranking (listed in descending order of robustness).

#### 4.2.2. Analysis of Experimental Results for Squared-Type Operators

From the experimental results, it can be seen that for the same image sequence, the noise response slopes of different operators differ significantly, yet their numerical values remain comparable. For different image sequences, the noise response slope of the same operator varies markedly, indicating that the noise response slope changes with image content, but the robustness ranking of the operators remains unchanged. For all image sequences, the Spearman correlation coefficient between the theoretical slope and the experimental slope is 1, with an exact probability of *p* = 0.017 (two-tailed), which is significant at the 0.05 level. Moreover, the results of repeated experiments show that the standard deviation of Re for each operator is much smaller than its mean, indicating that random noise has limited impact on the overall conclusions, and the proposed model exhibits good stability and repeatability.

Except for the sine image sequence, the relative errors between the theoretical slope and the experimental slope for the other image sequences remain low, with a maximum relative error not exceeding 2%. For the sine image sequence, there is a noticeable difference (less than 10%) between the values of *k_theory_* and *k_exp_*_1_, but the difference between *k_theory_* and *k_exp_*_2_ is very small (less than 2%), and the Re1 values of different operators are essentially the same. This indicates that the error is mainly caused by the image itself and has little relation to the computational structure of the focus measure operators. The sine image contains a large number of pixels with grayscale values near the extremes (0 or 255). Grayscale truncation significantly affects the noise distribution, leading to a considerable deviation in the *k_exp_*_1_ values. Comparing the experimental data under the two grayscale processing conditions shows that the influence of grayscale truncation on the noise response model proposed in this paper depends primarily on the grayscale distribution of the image. If the image contains many pixels with grayscale values near the extremes, a notable deviation occurs; if most pixels have grayscale values at intermediate levels, grayscale truncation does not have a significant effect. In practical applications, the grayscale distribution of most images is at intermediate levels, and under such conditions, the proposed noise response model exhibits strong robustness against deviations in statistical distribution caused by limited dynamic range. For the sine image sequence, although there is a noticeable difference between *k_theory_* and *k_exp_*_1_, the robustness ranking of operators based on *k_exp_*_1_ values is the same as that based on *k_theory_* and *k_exp_*_2_ values.

Overall, for squared-type operators, the theoretical model established based on the assumption of noise energy superposition can accurately characterize the error propagation behavior under noise, and it is therefore feasible to use *k_theory_* as an indicator for evaluating operator robustness.

#### 4.2.3. Linear Validation for Absolute-Value-Type Operators

**Figure 6:**
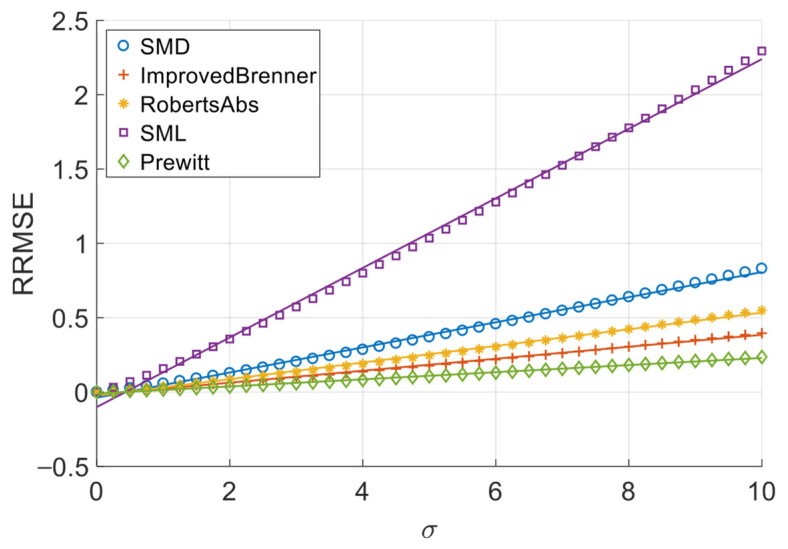
*RRMSE*–*σ* experimental values and their fitted lines under the Cone sequence.

**Figure 7:**
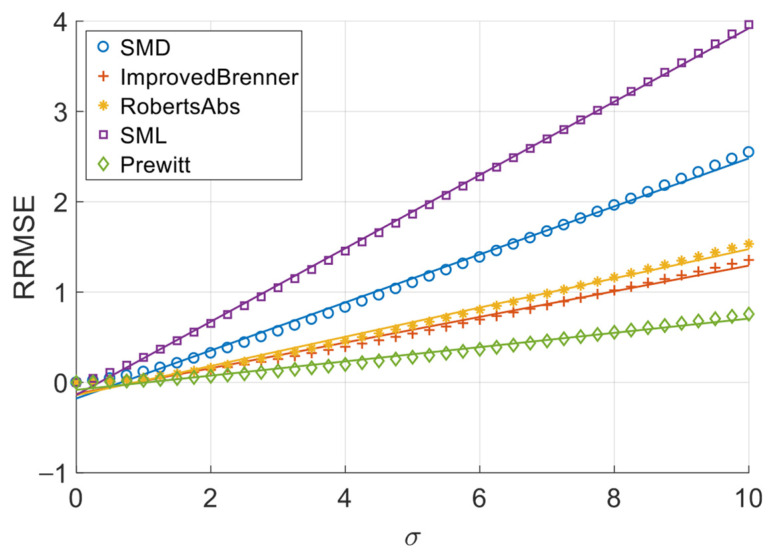
*RRMSE*–*σ* experimental values and their fitted lines under the Sine sequence.

**Figure 8:**
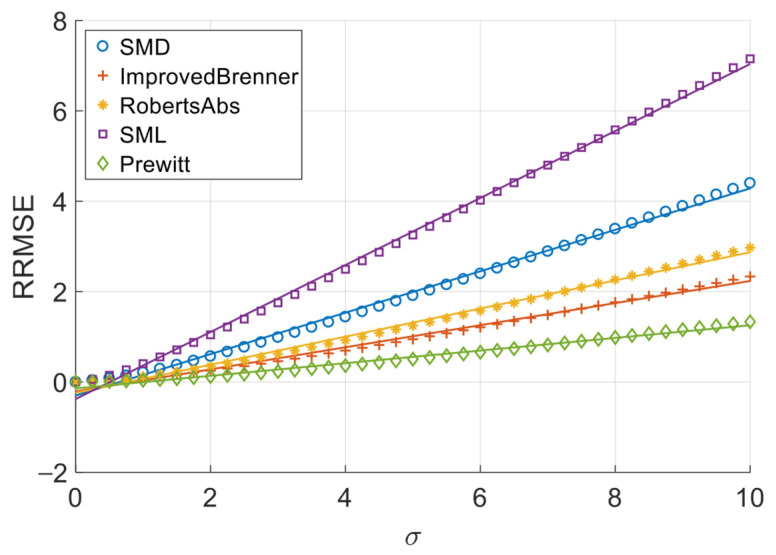
*RRMSE*–*σ* experimental values and their fitted lines under the Coin sequence.

**Figure 9:**
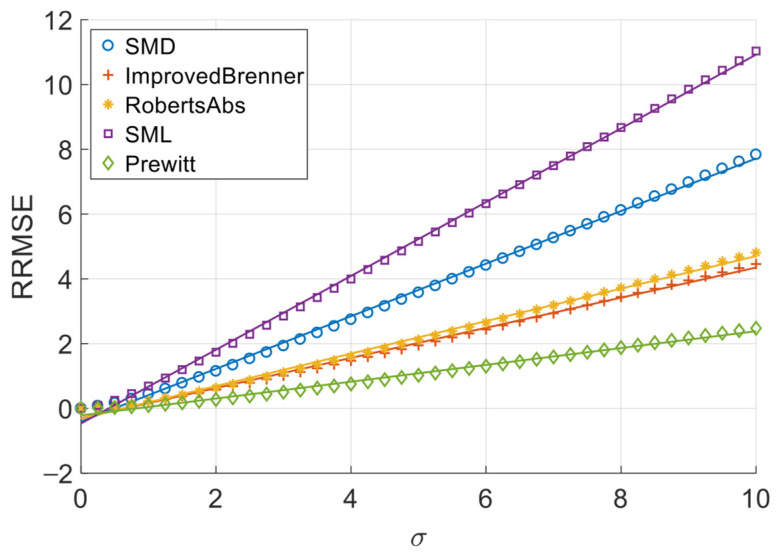
*RRMSE*–*σ* experimental values and their fitted lines under the Pencil sequence.

Figure 6, Figure 7, Figure 8 and Figure 9 illustrate the single-experiment values and fitted lines of *RRMSE* versus *σ* for the five absolute-value-type operators introduced in Chapter 3 under different image sequences.

From Figure 6, Figure 7, Figure 8 and Figure 9, it is observed that for all sequences, the *RRMSE* of each absolute-value-type operator generally increases monotonically with *σ*. Unlike square-type operators, the *RRMSE*–*σ* curves exhibit some nonlinear deviation in the low-noise region, gradually approaching linearity as noise intensity increases. The fitted lines show negative intercepts, which is consistent with the theoretical model.

To quantitatively analyze the overall noise response characteristics of absolute-value-type operators, the linear fit goodness (*R*^2^) over the entire noise range was calculated, as shown in Table 8. It can be seen that the linear goodness of fit of each operator under different image sequences is maintained at a high level as a whole, and the *R*^2^ values are all greater than 0.98, indicating that the relationship between *RRMSE* and noise standard deviation sigma can be approximated by a linear model.

The experimental results demonstrate that when noise variance is small, the slope of the *RRMSE*-*σ* curve exhibits significant variation with changes in noise variance; however, when noise variance is large, the slope remains nearly constant. Clearly, when noise variance is sufficiently small, the area of grayscale flat areas approaches zero, while the area of grayscale change areas approaches the total image area. As previously discussed, the *RRMSE* output approaches zero under these conditions. As noise variance increases, the area of grayscale flat areas gradually expands, while the area of grayscale change areas decreases—resulting in an increasing *p*-value. This leads to a gradual rise in the slope of the *RRMSE*-*σ* curve, indicating a noticeable change in its gradient. When noise variance reaches a certain threshold, further increases in noise variance no longer significantly alter the area of grayscale flat areas or the *p*-value; consequently, the slope of the *RRMSE*-*σ* curve remains largely unchanged, approximating a straight line. Therefore, in noise-dominated high-noise regions, the *RRMSE* of absolute-value operators is approximately proportional to the noise standard deviation σ, allowing the noise response slope to serve as an indicator of their robustness. Equations (20) and (28) objectively describe this phenomenon. In practical applications, we recommend using the noise response slope to estimate the robustness of absolute-value operators when the noise standard deviation exceeds 1.

Differences in fit results across sequences are related to the structural complexity of image content, but they do not change the basic linear trend. This indicates that the proposed noise response model is applicable across different types of image sequences.

Table 9, Table 10, Table 11 and Table 12 present, for five absolute-value type operators on different image sequences, the theoretical slope *k_theory_*, as well as the mean values of the experimental slopes *k*_*exp*1_ (Restricted grayscale range) and *k*_*exp*2_ (Unrestricted grayscale range) obtained from repeated experiments under the two grayscale processing conditions, together with the mean and standard deviation of the corresponding relative errors Re_1_ and Re_2_.

#### 4.2.4. Analysis of Experimental Results for Absolute-Value-Type Operators

It can be seen from the experimental results that the deviation between *k_theory_* and *k_exp_* of the absolute value operator is large. Overall, the deviation on the simulated image sequence is relatively large, and the deviation on the real image sequence is relatively small. It can be seen from the previous text that in addition to the grayscale flat area and the grayscale obvious change area, there is also a grayscale transition area in the image. The theoretical model in this paper ignores the grayscale transition area, so there is an error. Although there are differences between the two, *k_theory_* is always greater than *k_exp_*, which is consistent with the assumption of this paper.

For the same image sequence, the relative errors of *k_theory_* and *k_exp_* of different absolute value operators are different, mainly because the noise robustness of different operators is different, which leads to the different division of the grayscale flat area, grayscale transition area and grayscale obvious area of the image by the operator. That is, the grayscale flat area for one operator belongs to the grayscale obvious area or grayscale transition area for another operator. Therefore, the division of the grayscale area of the image is not fixed, but is related to the robustness of the operator. It can be seen from the experimental results that the stronger the robustness of the operator, the greater the relative error, and the weaker the robustness of the operator, the smaller the relative error.

At the same time, it can be seen from the experimental results that the relative error of the same absolute value operator is different for different image sequences. For example, the relative error of the Prewitt operator in the cone image sequence is 42.08%, and the relative error in the Pencil image sequence is only 18.98%, which shows that the relative error is also closely related to the structural complexity of the image content. Therefore, for the absolute value operator, the relative error of *k_theory_* and *k_exp_* is related to both the image content and the operation structure of the operator. To estimate the actual value of the noise response slope more accurately in practical applications, we recommend multiplying the theoretical slope by a coefficient. This coefficient is derived from the mean Re value and appropriately approximated: for synthetic image sequences, the coefficient is 0.7; for real image sequences, the coefficient is 0.85.

Similar to square-type operators, grayscale truncation has a significant impact on the experimental values for the sine image sequence (*k_exp_*_1_ values are noticeably larger than *k_exp_*_2_ values), while its effect on other image sequences is minor. This further validates our inference—that the deviation is caused by the large number of pixels at the grayscale extremes in the sine image sequence and is unrelated to the computational structure of the operators.

For the absolute-valued focusing evaluation operator, although there is always a certain difference between *k_theory_* and *k_exp_*, there is a certain law in this difference, that is, the relative errors of all operators change synchronously. For example, for all operators, the relative error of the Pencil image sequence is always the smallest, while the relative error of the cone image sequence is always the largest, and it also shows a similar law on the sine image sequence and the coin image sequence. In addition, we have also done experiments on other image sequences, all of which show similar laws, which shows that the change law of the relative error is mainly determined by the image content. As a result, although there are differences between *k_theory_* and *k_exp_*, the order of operator robustness is exactly the same for different image sequences. For all image sequences, the Spearman correlation coefficient between theoretical slope and experimental slope was 1, with an exact probability of *p* = 0.017 (two-tailed), which is significant at the 0.05 level. For operator robustness analysis, the main purpose is to compare the robustness of different operators and provide a reference for operator selection. From this perspective, *k_theory_* can be used as an upper limit estimate of the actual value of the noise response slope and as a reference index for evaluating the robustness of absolute-valued operators.

Combining the results for square-type and absolute-value-type operators, the theoretical sensitivity coefficient *k_theory_* numerically approximates *k_exp_* well enough to capture the basic characteristics of the *RRMSE* growth rate under noise. This means that, in practical applications, one can estimate the noise sensitivity of an operator using image statistics and operator structural parameters without performing multiple noise-addition experiments. Compared with computing *RRMSE* at a specific noise level, the noise response slope, as a parameter describing the error trend with noise, does not depend on the choice of noise intensity and more intuitively reflects the overall noise sensitivity of the operator. Therefore, the noise response slope provides a more concise analysis tool for selecting and optimizing focus measure operators.

## 5. Conclusions

This paper systematically analyzes the noise robustness of focus measure operators from both theoretical derivation and experimental validation, and establishes a quantitative relationship model between the *RRMSE* and noise parameters. On this basis, a new robustness metric—the noise response slope—is proposed, providing a quantitative reference for evaluating the robustness of operators. The study reveals the essential differences in error growth mechanisms between different types of operators under AWGN. Theoretical analysis and experimental results show that:

1. For squared-type operators, the *RRMSE* is highly linearly correlated with the noise variance *σ*^2^ across all tested sequences. When the image gray-level distribution lies in the mid-range, the theoretical slope and the mean experimental regression slope are in high agreement; when the image contains a large number of pixels at the extreme gray limits, a deviation exists between the theoretical slope and the mean experimental regression slope. This deviation is typically less than 10% and does not alter the robustness ranking of the operators.
2. For absolute-value type operators, the *RRMSE* is approximately linearly correlated with the noise standard deviation *σ* over the full noise range, with a certain nonlinear deviation when the noise variance is small, which is consistent with the theoretical assumption of “large noise variance”. Although there is a numerical difference between the theoretical slope and the experimental slope, their rankings of operator robustness are consistent.

The proposed “noise response slope” describes the global trend of *RRMSE* as a function of noise intensity. This metric can be directly calculated via theoretical formulas without actually adding noise to the image sequences. Overall, this metric can reveal the intrinsic sensitivity of operators to noise and provide a theoretical benchmark for robustness evaluation. The theoretical model presented in this paper provides a more concise and efficient theoretical analysis tool for performance evaluation of focus measure operators and even for the design of new operators, and holds good potential for engineering applications. For absolute-value type operators, there still exists a noticeable deviation between the theoretical and experimental values, and the theoretical model requires further optimization. Future work can extend the model to other noise types (e.g., Poisso–Gaussian mixed noise) and introduce finer modeling of gray-level transition regions to reduce the theoretical deviation of absolute-value type operators, thereby enhancing its applicability in practical focus measurement and 3D surface reconstruction systems.

## Figures and Tables

**Table 1:** Expressions of *k_theory_* for different operator types.

Operator Type	k_theory_
Neighborhood-difference Square-type	$k_{theory}=\frac{2n}{\mu }$
Convolution Square-type	$k_{theory}=\frac{1}{\mu }\left[\sum_{u,v}H^{2}\left(u,v\right)\right]$
Neighborhood-Difference Absolute-Value-Type	$k_{theory}=\frac{2n}{\mu \sqrt{\pi }}$
Convolution Absolute-Value-Type	$k_{theory}=\frac{1}{\mu }\sqrt{\frac{2}{\pi }\sum_{u,v}H^{2}(u,v)}$

**Table 2:** Parameters of image sequences.

Parameter	Cone	Sine	Coin	Pencil
Number of images	90	90	55	80
Image size (pixels)	360 × 360	256 × 256	1303 × 1356	512 × 512

**Table 3:** Goodness of Linear Fit of Squared-Type Operators on Different Sequences.

FM	Cone (*R*^2^)	Sine (*R*^2^)	Coin (*R*^2^)	Pencil (*R*^2^)
Brenner	1.0000	0.9998	1.0000	1.0000
EOG	1.0000	0.9999	1.0000	1.0000
Roberts	1.0000	0.9999	1.0000	1.0000
Tenengrad	1.0000	0.9998	1.0000	1.0000
EOL	1.0000	0.9998	1.0000	1.0000

**Table 4:** Slope Comparison of Squared-Type Operators on the Cone Sequence.

FM	k_theory_	k_exp1_	k_exp2_	Re_1_ (%)	Re_2_ (%)	S_1_	S_2_
Brenner	0.0018(2)	0.0018(2)	0.0018(2)	0.6410	0.5864	0.0456	0.0337
EOG	0.0064(4)	0.0064(4)	0.0064(4)	0.3083	0.2833	0.0110	0.0227
Roberts	0.0030(3)	0.0030(3)	0.0030(3)	0.3300	0.5648	0.0185	0.0239
Tenengrad	0.0007(1)	0.0007(1)	0.0007(1)	0.5183	1.1581	0.1026	0.0713
EOL	0.0332(5)	0.0331(5)	0.0331(5)	0.0700	0.0526	0.0088	0.0282

**Table 5:** Slope Comparison of Squared-Type Operators on the Sine Sequence.

FM	k_theory_	k_exp1_	k_exp2_	Re_1_ (%)	Re_2_ (%)	S_1_	S_2_
Brenner	0.0128(2)	0.0117(2)	0.0127(2)	9.2658	0.8366	0.0307	0.0314
EOG	0.0261(4)	0.0238(4)	0.0260(4)	8.7855	0.4451	0.0107	0.0279
Roberts	0.0176(3)	0.0160(3)	0.0174(3)	9.2206	0.8743	0.0063	0.0251
Tenengrad	0.0071(1)	0.0064(1)	0.0070(1)	9.0496	1.5663	0.0400	0.0353
EOL	0.0300(5)	0.0275(5)	0.0300(5)	8.4470	0.1459	0.0127	0.0115

**Table 6:** Slope Comparison of Squared-Type Operators on the Coin Sequence.

FM	k_theory_	k_exp1_	k_exp2_	Re_1_ (%)	Re_2_ (%)	S_1_	S_2_
Brenner	0.0571(2)	0.0570(2)	0.0570(2)	0.15995	0.1545	0.0035	0.0082
EOG	0.1418(4)	0.1417(4)	0.1417(4)	0.07497	0.0774	0.0039	0.0036
Roberts	0.0822(3)	0.0820(3)	0.0820(3)	0.15410	0.1526	0.0020	0.0026
Tenengrad	0.0242(1)	0.0241(1)	0.0241(1)	0.31590	0.3001	0.0058	0.0037
EOL	0.2981(5)	0.2980(5)	0.2980(5)	0.01207	0.0174	0.0062	0.0046

**Table 7:** Slope Comparison of Squared-Type Operators on the Pencil Sequence.

FM	k_theory_	k_exp1_	k_exp2_	Re_1_ (%)	Re_2_ (%)	S_1_	S_2_
Brenner	0.1275(2)	0.1264(2)	0.1270(2)	0.8068	0.3888	0.0043	0.0114
EOG	0.3496(4)	0.3475(4)	0.3489(4)	0.5845	0.1895	0.0066	0.0076
Roberts	0.1910(3)	0.1895(3)	0.1903(3)	0.7878	0.3847	0.0090	0.0053
Tenengrad	0.0542(1)	0.0535(1)	0.0537(1)	0.2252	0.7857	0.0117	0.0161
EOL	0.6717(5)	0.6688(5)	0.6714(5)	0.4217	0.0318	0.0072	0.0114

**Table 8:** Goodness of Linear Fit of Absolute-Value-Type Operators on Different Sequences.

FM	Cone (*R*^2^)	Sine (*R*^2^)	Coin (*R*^2^)	Pencil (*R*^2^)
SMD	0.9969	0.9961	0.9955	0.9981
ImprovedBrenner	0.9970	0.9902	0.9909	0.9960
RobertsAbs	0.9969	0.9923	0.9931	0.9965
SML	0.9976	0.9995	0.9979	0.9990
Prewitt	0.9971	0.9827	0.9852	0.9926

**Table 9:** Slope Comparison of Absolute-Value-Type Operators on the Cone Sequence.

FM	k_theory_	k_exp1_	k_exp2_	Re_1_ (%)	Re_2_ (%)	S_1_	S_2_
SMD	0.1229(4)	0.0844(4)	0.0845(4)	31.2667	31.2599	0.0088	0.0043
ImpBrenner	0.0656(2)	0.0402(2)	0.0402(2)	38.6075	38.6081	0.0058	0.0032
RobertsAbs	0.0856(3)	0.0586(3)	0.0586(3)	34.7283	34.7244	0.0061	0.0052
SML	0.2870(5)	0.2340(5)	0.2340(5)	18.4425	18.4371	0.0043	0.0049
Prewitt	0.0414(1)	0.0240(1)	0.0240(1)	42.0836	42.0891	0.0082	0.0079

**Table 10:** Slope Comparison of Absolute-Value-Type Operators on the Sine Sequence.

FM	k_theory_	k_exp1_	k_exp2_	Re_1_ (%)	Re_2_ (%)	S_1_	S_2_
SMD	0.3157(4)	0.2477(4)	0.2658(4)	21.5096	15.7765	0.0071	0.0115
ImpBrenner	0.1923(2)	0.1310(2)	0.1479(2)	31.8559	26.1651	0.0050	0.0115
RobertsAbs	0.2372(3)	0.1736(3)	0.1871(3)	26.8241	21.1310	0.0033	0.0110
SML	0.4431(5)	0.3821(5)	0.4059(5)	13.7721	8.3970	0.0100	0.0113
Prewitt	0.1248(1)	0.0725(1)	0.0789(1)	41.8702	36.7528	0.0144	0.0078

**Table 11:** Slope Comparison of Absolute-Value-Type Operators on the Coin Sequence.

FM	k_theory_	k_exp1_	k_exp2_	Re_1_ (%)	Re_2_ (%)	S_1_	S_2_
SMD	0.5232(4)	0.4585(4)	0.4585(4)	12.3796	12.3782	0.0019	0.0011
ImpBrenner	0.3088(2)	0.2445(2)	0.2445(2)	20.8086	20.8072	0.0013	0.0026
RobertsAbs	0.3876(3)	0.3223(3)	0.3223(3)	16.8287	16.8283	0.0023	0.0013
SML	0.8003(5)	0.7415(5)	0.7415(5)	7.3496	7.3485	0.0020	0.0017
Prewitt	0.1995(1)	0.1398(1)	0.1398(1)	29.9354	29.9351	0.0035	0.0032

**Table 12:** Slope Comparison of Absolute-Value-Type Operators on the Pencil Sequence.

FM	k_theory_	k_exp1_	k_exp2_	Re_1_ (%)	Re_2_ (%)	S_1_	S_2_
SMD	0.8730(4)	0.8102(4)	0.8119(4)	7.1843	6.9936	0.0047	0.0063
ImpBrenner	0.5291(2)	0.4640(2)	0.4650(2)	12.3047	12.1128	0.0064	0.0054
RobertsAbs	0.6122(3)	0.5467(3)	0.5479(3)	10.6834	10.4890	0.0033	0.0055
SML	1.1915(5)	1.1356(5)	1.1387(5)	4.6109	4.4299	0.0033	0.0072
Prewitt	0.3198(1)	0.2591(1)	0.2598(1)	18.9790	18.7744	0.0084	0.0043

## Data Availability

The data that support the findings of this study are available from the corresponding author upon reasonable request.
